# Desiccation tolerance in the resurrection plant *Barbacenia graminifolia* involves changes in redox metabolism and carotenoid oxidation

**DOI:** 10.3389/fpls.2024.1344820

**Published:** 2024-02-15

**Authors:** Evandro Alves Vieira, Marilia Gaspar, Cecílio Frois Caldeira, Sergi Munné-Bosch, Marcia Regina Braga

**Affiliations:** ^1^ Vale Institute of Technology, Belém, Brazil; ^2^ Biodiversity Conservation Center, Institute of Environmental Research, São Paulo, Brazil; ^3^ Department of Evolutionary Biology, Ecology, and Environmental Sciences, Faculty of Biology, University of Barcelona, Barcelona, Spain

**Keywords:** apocarotenoids, β-carotene, β-cyclocitral, photoprotection, phytohormone signaling, reactive-oxygen species, tocopherols

## Abstract

Desiccation tolerance in vegetative tissues enables resurrection plants to remain quiescent under severe drought and rapidly recover full metabolism once water becomes available. *Barbacenia graminifolia* is a resurrection plant that occurs at high altitudes, typically growing on rock slits, exposed to high irradiance and limited water availability. We analyzed the levels of reactive oxygen species (ROS) and antioxidants, carotenoids and its cleavage products, and stress-related phytohormones in fully hydrated, dehydrated, and rehydrated leaves of *B. graminifolia*. This species exhibited a precise adjustment of its antioxidant metabolism to desiccation. Our results indicate that this adjustment is associated with enhanced carotenoid and apocarotenoids, α-tocopherol and compounds of ascorbate-glutathione cycle. While α-carotene and lutein increased in dried-leaves suggesting effective protection of the light-harvesting complexes, the decrease in β-carotene was accompanied of 10.2-fold increase in the content of β-cyclocitral, an apocarotenoid implicated in the regulation of abiotic stresses, compared to hydrated plants. The principal component analysis showed that dehydrated plants at 30 days formed a separate cluster from both hydrated and dehydrated plants for up to 15 days. This regulation might be part of the protective metabolic strategies employed by this resurrection plant to survive water scarcity in its inhospitable habitat.

## Introduction

Desiccation tolerance (DT) is the ability of an organism to survive severe drought, losing up to 95% of subcellular water and fully recovering its metabolism after increased water availability. This phenomenon has been observed across various organisms, from bacteria to angiosperms (Farrant et al., 2003; Costa et al., 2017). Despite the diverse geographic distribution and taxonomic variety of resurrection plants, they share the common feature of full DT in their vegetative tissues, remaining dead-like after prolonged drought periods and reviving a few hours after rehydration (Farrant et al., 2007; Farrant et al., 2012; Vieira et al., 2017a; Vieira et al., 2017b; Tebele et al., 2021). In recent decades, resurrection plants have emerged as models to understand how a highly efficient protection system has evolved to enable them to cope with extreme drought without inducing irreversible damage to their tissues. Learning about these survival strategies is important for improving crop drought tolerance and food security under ongoing global climate changes (Radermacher et al., 2019).

Resurrection plants exhibit a range of both common and distinct strategies to cope with high irradiance and survive extreme dehydration (Gechev et al., 2021; Marks et al., 2021). Strategies such as shutdown of photosynthetic metabolism and degradation of chlorophylls prove effective in preventing photooxidative damage (Georgieva et al., 2005; Aidar et al., 2014; Rakić et al., 2015; Vieira et al., 2022). This is crucial as desiccation-induced imbalances in the carbon assimilation led to an increased production of reactive oxygen species (ROS) (Beckett et al., 2012; Tan et al., 2017). Elevated enzymatic antioxidant defenses and the accumulation of protective metabolites, such as ascorbate (vitamin C), tocopherols and tocotrienols (vitamin E), carotenoids, and oxidized and reduced glutathione, are identified as primary mechanisms for ROS scavenging in plants and green algae facing water scarcity (Pintó-Marijuan and Munné-Bosch, 2013; Griffiths et al., 2014; Vieira et al., 2017a; Radermacher et al., 2019; Moura and Vieira, 2020; Vieira et al., 2021; Roach et al., 2022a; Roach et al., 2022b).

Carotenoids are isoprenoid-derived lipophilic compounds that serve as essential photosynthetic pigments in plants, quenching excess light energy and scavenging ROS generated during the photosynthesis (Sierra et al., 2022). In chloroplasts, photooxidation under stress conditions is the main carotenoid- degrading process and produces apocarotenoids (Koschmieder et al., 2021). It involves the oxidative cleavage of carotenoids, catalyzed by dioxygenases (CCDs) or exposure to ROS (Felemban et al., 2019). The oxidative cleavage of the β-carotene polyene produces a volatile short-chain compound known as β-cyclocitral (β-CC) (D’Alessandro and Havaux, 2019). In Arabidopsis, the pre-treatment of plants with β-CC resulted in the accumulation of β-cyclocitric acid (β-CCA), enhancing drought tolerance and photoprotection by reducing lipid peroxidation and PSII photoinhibition (D'Alessandro et al., 2019).

The essential antioxidant role of carotenoids in protecting the photosynthetic apparatus during dehydration has been previously reported in resurrection plants such as *Haberlea rhodopensis* (Rapparini et al., 2015), Ramonda serbica and R. nathaliae (Rakić et al., 2015), *R. micony* (Fernandéz-Marín et al., 2020), *Pilea microphylla* (Greeshma and Murugan, 2015), and *Boea hygrometrica* (Mitra et al., 2013). However, the potential role of the high degradation of carotenoids and their oxidation products in the late drying stages is still poorly understood. In the DT species *Eragrostis nindensis*, the genes for carotenoid biosynthesis are more highly expressed than in *E. tef*, a desiccation-sensitive species (Pardo et al., 2020). Some authors (Peñuelas and Munné-Bosch, 2005; Ramel et al., 2012) have suggested that carotenoid oxidized metabolites may have a potential protective function in sensing and signaling oxidative stress conditions and energy dissipation.

Another well-known apocarotenoid is abscisic acid (ABA), a phytohormone essential in drought resistance, stomata closure regulation, sugar sensing, and interaction with other phytohormones (Felemban et al., 2019). ABA biosynthesis starts with the CCD-mediated enzymatic cleavage of 9-cis-violaxanthin or 9’-cis-neoxanthin into xanthoxin and the corresponding C_25_-apocarotenoid in plastids. Xanthoxin is then transported to the cytosol, and short-chain alcohol dehydrogenase converts xanthoxin to abscisic aldehyde, which abscisic aldehyde oxidase oxidizes to ABA (Moreno et al., 2021). Along with ABA, jasmonates have been implicated in promoting stomatal closure and modulating root hydraulic conductivity, contributing to soil water uptake under water deficit (Villadangos and Munné-Bosch, 2023). In the leaves and roots of the resurrection plant *H*. *rhodopensis*, ABA and jasmonic acid (JA) serve as signals that trigger the desiccation tolerance response (Djilianov et al., 2013). Similarly, the accumulation of ABA-regulated transcripts is also an early response to dehydration in the desiccation-tolerant *Craterostigma plantagineum* (Rodriguez et al., 2010). However, the mechanisms of ABA formation from the oxidation of carotenoids in resurrection plants are not yet fully elucidated.

Malondialdehyde (MDA) is another compound widely used as an oxidative stress marker and induces the production of antioxidants that enable ROS control in plant cells (Muñoz and Munné-Bosch, 2020). In *Arabidopsis*, the heterologous expression of the MfWRY7 transcription factor of the resurrection plant *Myrothamnus flabillifolia* increased antioxidant protection in response to drought, reducing MDA content (Huang et al., 2022). Thus, MDA may benefit from activating regulatory genes involved in defense and development (Morales and Munné-Bosch, 2019).

*Barbacenia graminifolia* L. B. Sm. is a resurrection plant to “Campos Rupestres” in Serra do Cipó, Minas Gerais state (Brazil). It occurs at high altitudes (800–1700 m), typically growing on rock slits, exposed to high irradiance and limited water availability (Mello-Silva et al., 2011). *Barbacenia graminifolia* exhibits morphological, physiological, and metabolic adaptations to cope with desiccation. It has differentiated strategy considering a time frame of responses to survive to severe water scarcity. Despite degrading photosynthetic pigments and turning off the photochemical metabolism under drastic water suppression, it sustains hydration levels much longer during water shortage than other resurrection species of *Barbacenia* (Suguiyama et al., 2014; Nascimento et al., 2020; Vieira et al., 2022). This water loss behavior of *B. graminifolia* makes it a suitable model to study the diversity of physiological and metabolic responses in resurrection plants to overcome stressful environments.

In this study, we investigated how levels of non-enzymatic antioxidants, carotenoids, apocarotenoids, and stress-related phytohormones vary during *B*. *graminifolia* dehydration and re-hydration processes. We hypothesized that changes in α-tocopherol and compounds of ascorbate-glutathione cycle, together with carotenoid degradation and accumulation of their oxidation products, occur as part of the response of this species to water scarcity.

## Materials and methods

### Plant material and experimental design

Resurrection plants of *Barbacenia graminifolia* L. B. Sm. were collected from a *campos ruspestres* in Serra do Cipó National Park (Minas Gerais, Brazil; 19° 12’ and 19° 34’ south, 43° 27’ and 43° 38’ west). Plants were transplanted into 3L pots containing commercial substrate (Plantmax^®^) and kept in a greenhouse of the Biodiversity Conservation Center, Institute of Environmental Research, São Paulo, Brazil. They were watered with half-strength Hoagland’s nutrient solution (Hoagland and Arnon, 1950) and kept under controlled conditions for acclimation for 30 days. Afterwards, potted plants were subjected to the following treatments: 1) control, plants watered daily until field capacity, and 2) desiccation, plants subjected to irrigation suppression for 30 days, with subsequent rehydration. The experimental layout was completely randomized. The physiological and biochemical analyses were performed in samples collected at 0, 15 and 30 days of watering suppression and 24 and 120h after rehydration. Based on the experimental design from previous study (Vieira et al., 2022), we incorporated additional data points during desiccation and recovery phases to gain a more comprehensive understanding of the phenomenon. Analyses were conducted on five plants per treatment, utilizing the middle portion of two fully expanded leaves in identical positions in each plant. Soil moisture (%) was monitored daily throughout the experiment by time-domain reflectometry (TDR), using a soil moisture probe (ML2 x ThetaProbe) connected to an HH2 Moisture Meter (Delta-T Devices). Climatic data such as air temperature (°C), relative humidity (RH%), and the photosynthetic photon flux density (PPFD, μmol photons m^−2^ s^−1^) were monitored inside the greenhouse using a temperature/humidity sensor (Li-1400-140 Li-Cor-Nebraska) and a quantum sensor (Li-190SA, Li-Cor Nebraska). The sensor was connected to a data logger (Li-1400; Li-Cor-Nebraska) that calculated daily means of each parameter evaluated. Leaf samples were collected and powdered in liquid nitrogen, and all biochemical analyses were performed in five replicates per treatment.

### Assessment of water status

The leaf relative water content (RWC) was evaluated in fresh leaves from five plants of each treatment. The leaves were weighed to obtain the fresh weight (FW), then hydrated with distilled water for 24h, and weighed again to obtain the turgid weight (TW). The dry weight (DW) was measured in oven-dried leaves at 65°C for 72 h. The RWC was calculated using the equation: (FW− DW)/(TW−DW) x 100. The osmotic potential (Ψs) was analyzed in the leaf sap extracted by overpressure in a pressure pump. Frozen leaf samples were defrosted at room temperature. The Ψs was measured in 10μl of each sample using a model 5520 vapor pressure osmometer (VAPRO).

### Oxidative injury indicators and redox potential

The superoxide anion (O_2_•^–^) production rate was measured by monitoring the nitrite formation from hydroxylamine in the presence of O_2_•^–^ (Wu et al., 2010). An aliquot (200 mg) of powdered leaves was mixed with 1.5 mL of 65 mM potassium phosphate buffer (pH 7.8) containing approximately 1% (w/v) polyvinylpolypyrrolidone (PVPP) and then centrifuged at 16,000 *g* (4°C, 25 min). For nitrite generation, aliquots of 125 µL of the supernatants were mixed with 125 µL of 65 mM potassium phosphate buffer (pH 7.8) and 25 µL of 10 mM hydroxylamine hydrochloride and then incubated at room temperature for 30 min, protected from light. Nitrite detection was performed by mixing equal parts of the incubated extract with Griess reagent, according to Antoniou et al. (2018). Absorbances were read at 540 nm in a microplate reader, and nitrite concentrations were calculated based on a sodium nitrite standard curve. The H_2_O_2_ content was estimated according to Sergiev et al. (1997). Powdered leaf samples (200 mg) were ground with 0.1% trichloroacetic acid and centrifuged at 12,000 *g* for 20 min. Then, a 300 μL aliquot was mixed with 300 μL phosphate-buffered saline (PBS; pH 7.0, 10 mM) and 800 μL of KCI (1 M). The H_2_O_2_ content was determined spectrophotometrically at 390 nm. The lipid peroxidation was indirectly determined by measuring the increase in malondialdehyde (MDA), according to Hodges et al. (1999). An aliquot of powdered leaves (80 mg) was homogenized with 1 ml of 80% ethanol and centrifuged at 3,000 *g* (4°C, 10 min). The supernatants were then incubated with 20% TCA and 0.01% hydroxytoluenebutylate, with or without 0.5% thiobarbituric acid (TBA), at 95°C for 25 min. Thereafter, samples were centrifuged as described above and the supernatant absorbance was measured at 440 nm, 532 nm (absorbance of specific MDA), and 600 nm (absorbance of non-specific MDA). The redox potential was measured by analyzing ascorbate (Asc), glutathione (GSH) and oxidized glutathione (GSSG) concentrations. Asc was determined in 100 mg of powdered leaves ground with 1.2 mL of 6% TCA previously frozen, according to Kampfenkel et al. (1995). Reduced (GSH) and oxidized glutathione (GSSG) fractions were quantified with the enzymatic recycling assay based on glutathione reductase. Total glutathione extraction was made with 100 mg of powdered leaves in 5% (w/v) sulfosalicylic acid (Israr et al., 2006). For GSSG assays, aliquots of 100 µL of the extracts were added to 900 µL of 0.5 mM sodium EDTA, 50 µL of 0.3 mM 5,5′dithio-bis-(2-nitrobenzoicacid) (DTNB) and 50 µL of 0.5 mM NADPH, all diluted in 100 mM potassium phosphate buffer (pH 7.0). Reactions were started with the addition of 1 µL of glutathione reductase. Mixtures were kept under light for 20 min. Absorbances were read in a spectrophotometer at 412 nm, and estimations were made based on a GSH standard curve. The GSH content was calculated by subtracting the GSSG from total glutathione content.

### Determination of carotenoid content

The extraction and analysis of carotenoids and xanthophylls were realized according to Kang et al. (2017). Five samples per treatment of powdered leaves (100 mg) were mixed with 5 mL of acetone containing 0.01% butylated hydroxytoluene (BHT), sea sand, Na_2_SO_4_, and NaHCO_3_. The extraction of carotenoids was conducted under low light to prevent degradation. The resultant extract was centrifuged at 5000 g (4°C, 5 min), and the supernatant was concentrated under vacuum and re-dissolved in CH_2_Cl_2_: acetone (1:1, 200 mL). The solution was filtered through a 0.45 mm membrane filter (Whatman, PTFE, 13 mm) and analyzed on an Agilent 1100 HPLC system (Hewlett-Packard). The standards or samples (20 µL) were injected directly onto a YMC C30 carotenoid column (3 µM, 4.6 x 250 mm) with solvent A (methanol: tert-butylmethyl ether (MTBE): H_2_O, 81:15:4, v/v) and solvent B (MeOH: MTBE: H_2_O, 6:90:4, v/v). A step-gradient elution of 100% solvent A was used for the first 15 min, followed by a gradient from 100% solvent A to 100% solvent B over the next 35 min. The eluent was detected at 450 nm on a UV-Vis detector. An external calibration method was used for carotenoid quantification. Standards of α-carotene, β-carotene, lutein, violaxanthin, antheraxanthin and zeaxanthin (Sigma-Aldrich) were used to compare peaks and the respective retention times. Carotenoid levels were expressed as the mean (µg/g dry weight) ± SD (standard deviation).

### Apocarotenoid analysis

Analysis of β-apo-13-carotenone, β-apo-8’-carotenal, β-apo-10’-carotenal, β-apo-12’-carotenal and β-apo-14’-carotenal levels was performed by LC-MS (Thermo Fisher Scientific) according to Schaub et al. (2018). Samples were ground to a fine powder in liquid nitrogen and mixed with an internal standard mix (75 pmol of each D3-β-apocarotenoid, Buchem) and 50 µg α-tocopheryl-acetate. Apocarotenoids were subjected to atmospheric pressure chemical ionization (APCI) and analyzed in positive ion mode. For quantification, the respective apocarotenoid compounds were used as internal standards. Standard curves were obtained with each unlabeled apocarotenoid in a range of 0.5−15 pmol on-column containing a 3 pmol constant amount of the respective D3-labelled compound. The TraceFinder 3.2 software was used for quantification based on the MS1 signal, with the MS2 spectra serving as a qualifier. Peak areas of the photometric signals at 285 nm were integrated for α-tocopheryl-acetate and used to correct for unspecific losses during sample processing. For glyoxal, methyl-glyoxal and β-cyclocitral determination, samples were dissolved in 300 µL dichloromethane and derivatized by adding 15 µL of 200 µM 2,4-dinitrophenylhydrazine phosphoric acid solution (DNPH, Sigma-Aldrich) and incubating at 37°C for 3 h. After washing with 1 mL of water, the organic phase was recovered, dried under vacuum, redissolved in 50 µL dichloromethane: methanol (1:1, v/v) and 2 µL were subjected to LC-MS analysis. The LC-MS parameters were the same as previously described, except that APCI ionization was performed in negative mode. Derivatization efficiency was monitored by measuring underivatized D3-labelled apocarotenoids. D3-β-apo-14’-carotenal-DNPH was used as internal standard for the normalization of apocarotene-dialdehyde signals.

### Tocopherol analysis

For α-tocopherol and γ-tocopherol concentration measurements, 50 mg of powdered leaves was subjected to three successive extractions in 3 mL of methanol: chloroform (2:1, v/v) containing butylated hydroxytoluene (0.01% w/v) as described by Yang et al. (2011). After 20 min of incubation, 1 mL of chloroform and 1.8 mL of water were added to each sample. After mixing and centrifuging, the lower organic layer was recovered, dried and resuspended in dichloromethane: methanol (1:5, v/v) for HPLC analysis; 5,7-dimethyltocol was added to each sample as an internal standard before extraction. The analysis was performed in an Agilent 1100 HPLC. Tocopherols were separated using an Agilent Eclipse XDB-C18 column (4.6 × 150 mm length; 5 µm particle size) and a solvent system consisting of methanol: water (95:5, v/v) with a 1.5 mL min^−1^ flow rate. Tocopherols in the sample were detected and quantified by fluorescence with excitation at 292 nm and emission at 330 nm, and the detector response factors were determined for each tocopherol species. Analyses were performed in five replicates per treatment. Quantification was based on the fluorescence signal and compared with a calibration curve with authentic standards (Sigma-Aldrich).

### Levels of stress-related phytohormones

The extraction and analysis of endogenous contents of abscisic acid (ABA), jasmonic acid (JA) and salicylic acid (SA) were carried out as described in Dobrev and Vankova (2012) and Djilianov et al. (2013). An aliquot (100 mg) of powdered leaves homogenized in liquid nitrogen was subjected to a cold extraction with buffer (500 µL) containing methanol/water/formic acid (15/4/1, v/v/v). Then, a mixture of stable isotope-labelled internal standards (10 pmol/sample): [^2^H_6_] ABA (Olchemin, Ltd); [^2^H_5_] JA (C-D-N Isotopes Inc.) and [^2^H_4_] SA (Sigma-Aldrich) were added to the plant homogenates. Reversed-phase and ion-exchange chromatography (Oasis-MCX, Waters) resulted in a fraction eluted with methanol. Fractions were evaporated to dryness in a vacuum concentrator and dissolved in 30 μL of 10% methanol. An aliquot (10 μL) of each fraction was analyzed separately by HPLC with Ultimate 3000 (Dionex, Sunnyvale) coupled to a hybrid triple quadrupole-linear ion trap mass spectrometer (3200 Q TRAP, Applied Biosystems) set to selected reaction-monitoring mode. The mass spectrometer was set at electrospray ionization mode, and the following ion source parameters were used: ion source voltage -4,000 V (negative mode); nebulizer gas 50 psi; heater gas 60 psi; curtain gas 20 psi; heater gas temperature 500°C. The phytohormones were quantified using the isotope dilution method with multilevel calibration curves and data expressed in ng/g dry weight.

### Statistical analysis

Drought stress was considered as a factor (independent variable), and the physiological and biochemical responses as variables (dependent variable). A completely randomized design was used, and all data were subjected to One-way ANOVA (p < 0.05) and compared by Tukey´s test using BioEstat version 5.3. The Principal Component Analysis (PCA) was conducted to discern the primary factors indicative of stress by examining the interrelation between physiological and biochemical parameters using the free web-based metabolomics tool MetaboAnalyst 6.0 (http://www.metaboanalyst.ca/).

## Results

### Effects of dehydration-rehydration on leaf water status and MDA content

**Figure 1:**
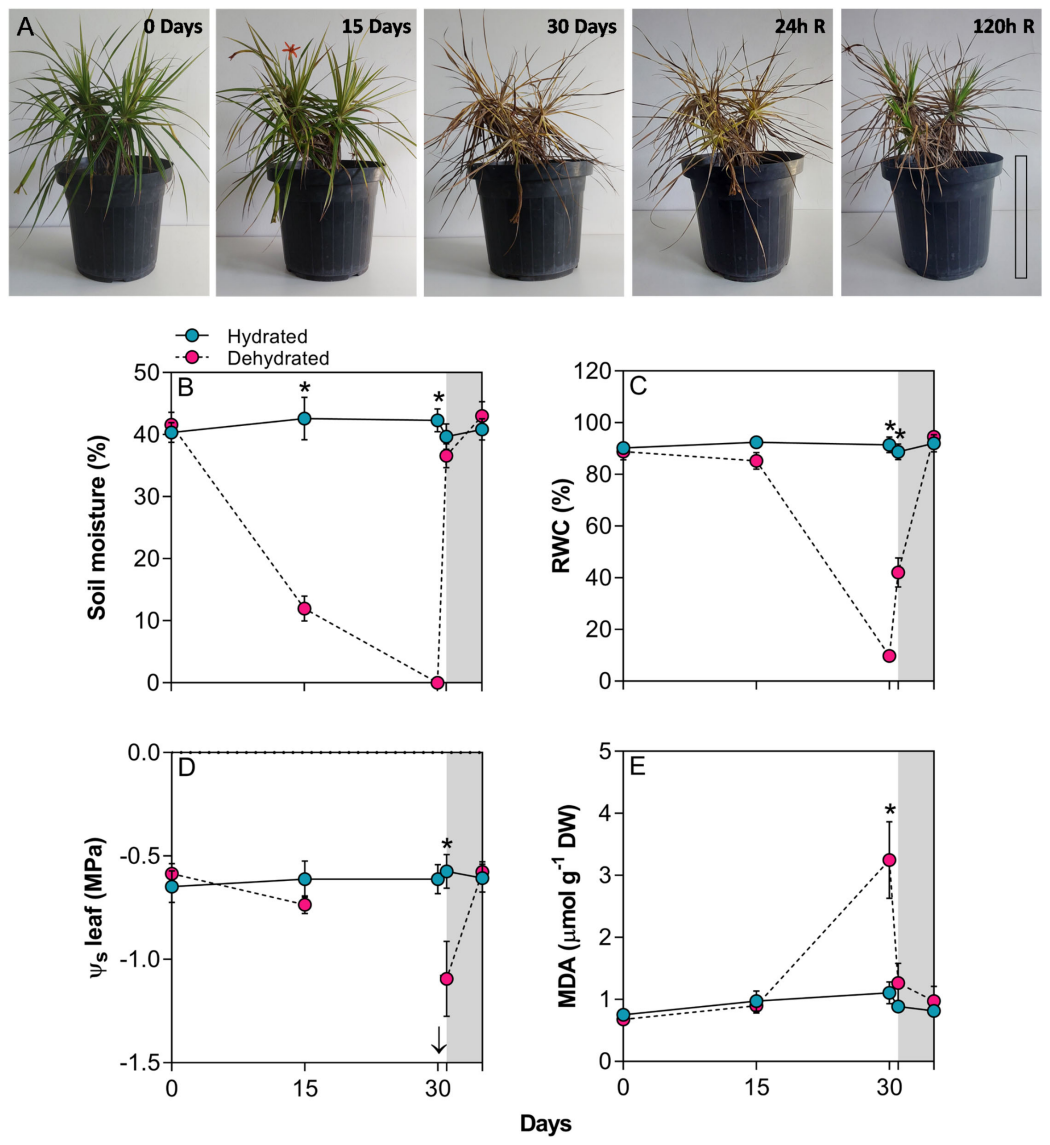
**(A)** General aspect of plants of *Barbacenia graminifolia* during the dehydration (0, 15 and 30 days) and after rehydration (24 and 120h). Scale = 20 cm. **(B)** Soil moisture (%), **(C)** leaf relative water content (RWC), **(D)** leaf osmotic potential (Ψs) and **(E)** malondialdehyde (MDA) content. The grey area in the figures represents the rehydration (between 30-35 days). The arrow indicates unmeasurable Ψs value in the desiccated tissue. *Significant difference between treatments at *P* < 0.05 (*n* = 5 ± *SD*). R = rehydration.

The imposition of water deficit in *B. graminifolia* induced typical morphological responses of resurrection plants, including a completely desiccated appearance, leaves curved inward towards the midrib, a drastic reduction in leaf area, and chlorophyll degradation (**Figure 1A**). Variations in the maximum temperature predominantly influenced the average temperature as the minimum temperature exhibit minimal changes over the experimental period (**Supplementary Figure 1A**). Relative air humidity (%) remained at around 59.7%, and the highest air temperature values coincided with high PPFD values. These conditions had an impact on the vapor pressure deficit (VPD), which remained constant between 30-35 days (**Supplementary Figure 1B**). The fluctuations in environmental conditions, coupled with the decline in soil water, contributed to the morphophysiological changes observed in *B*. *graminifolia* (**Figure 1A**).

The imposition of water stress significantly reduced leaf RWC at 30 days (**Figures 1B, C**). Although the decrease in RWC at 15 days reflected a declining trend in Ψs, observing a simultaneous decrease in both parameters at 30 days proved technically unfeasible to the impossibility to extract sap from the dehydrated leaves at 30 days. A significant difference in Ψs was observed 24 h after rehydration (p < 0.01) (**Figure 1D**). The dehydration process resulted in a 2.9-fold increase in MDA content at 30 days (**Figure 1E**). Following rehydration, the leaves began to recover their water status, initiating leaf expansion at 24h and restoring RWC and chlorophyll content close to control plant levels at 120h (**Figures 1A, C**). After rehydration, the level of MDA quickly dropped close to that of the control (**Figure 1D**).

### Redox adjustments

**Figure 2:**
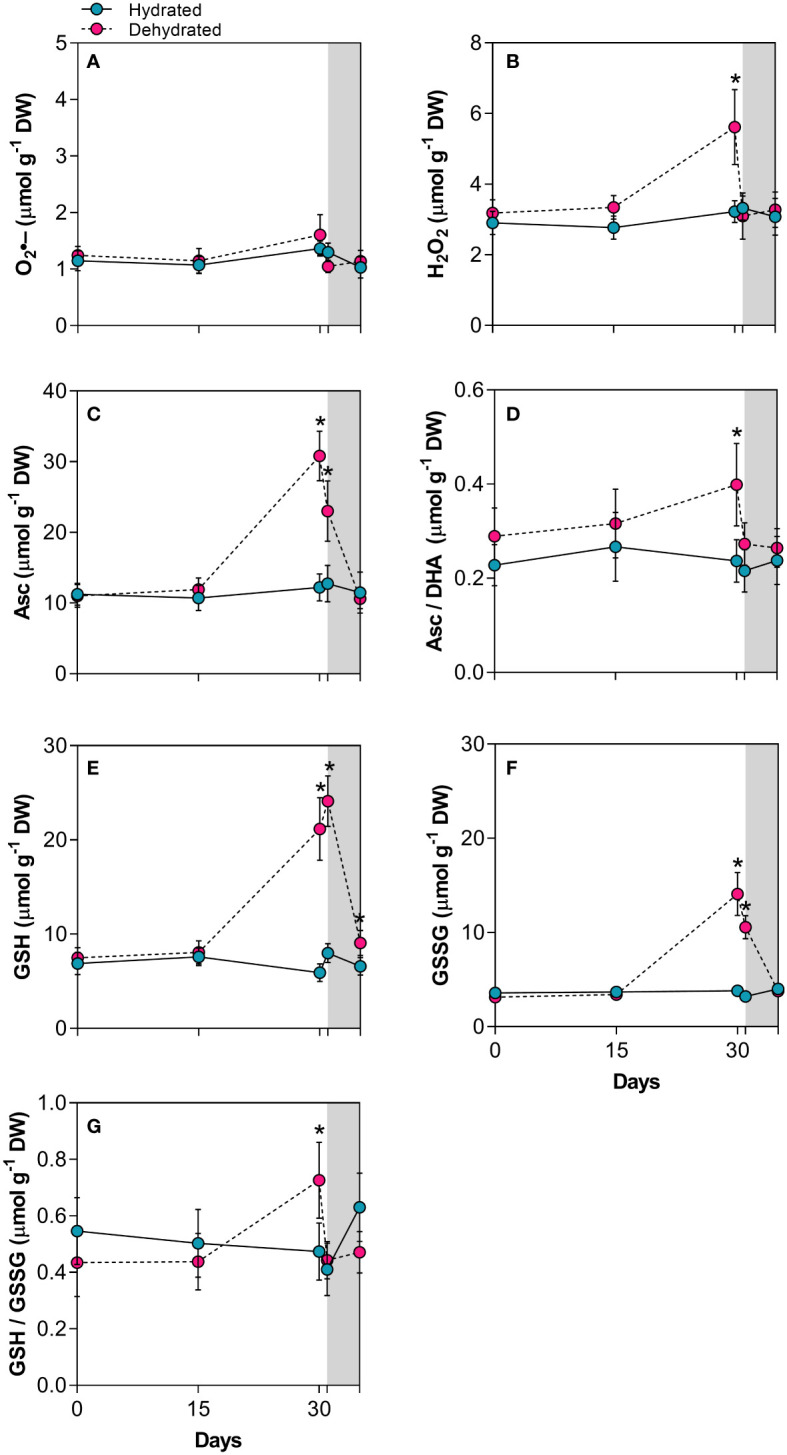
Indicators of oxidative injury and redox potential in leaves of *Barbacenia graminifolia* during dehydration (0, 15 and 30 days) and after rehydration (24 and 120h). Leaf content of **(A)** anion superoxide (O_2_•^-^), **(B)** hydrogen peroxide (H_2_O_2_), **(C)** ascorbate (Asc), **(D)** acorbate/dehydroascorbate ratio (Asc/DHA), **(E)** glutathione (GSH), **(F)** oxidized glutathione (GSSG) and **(G)** GSH/GSSG ratio. The grey area in the figure indicates the rehydration (between 30-35 days). *Significant difference between treatments at *P* < 0.05 (*n* = 5 ± *SD*).

The O_2_•^–^ content did not increase significantly during dehydration, indicating that *B*. *graminifolia* plants effectively control of this ROS (**Figure 2A**), considering short-lived species such as O_2_•^–^ and variations in their levels in the plant *in vivo*. In contrast, there was a 1.7-fold increase in H_2_O_2_ content (p < 0.01) at day 30, suggesting that these ROS and their effects are maintained at sublethal levels in desiccated leaves (**Figure 2B**). The increased levels of Asc, GSH and GSSG at 30 days of dehydration and 24 h after rehydration not only corroborated the action of these antioxidants during leaf drying but also reinforced the effective maintenance of these protective molecules during the early stages of rehydration, which are crucial for leaf restructuring (**Figures 2C, E, F**). The redox status (Asc/DHA) and GSH/GSSG also increased during the late desiccation stage, reaching significance only at 30 days (**Figures 2D, G**). Even 120h after rehydration, GSH levels remained high, while the other antioxidants and ROS returned to control levels (**Figure 2**).

### Carotenoid and xanthophyll content during the dehydration-rehydration cycle

**Figure 3:**
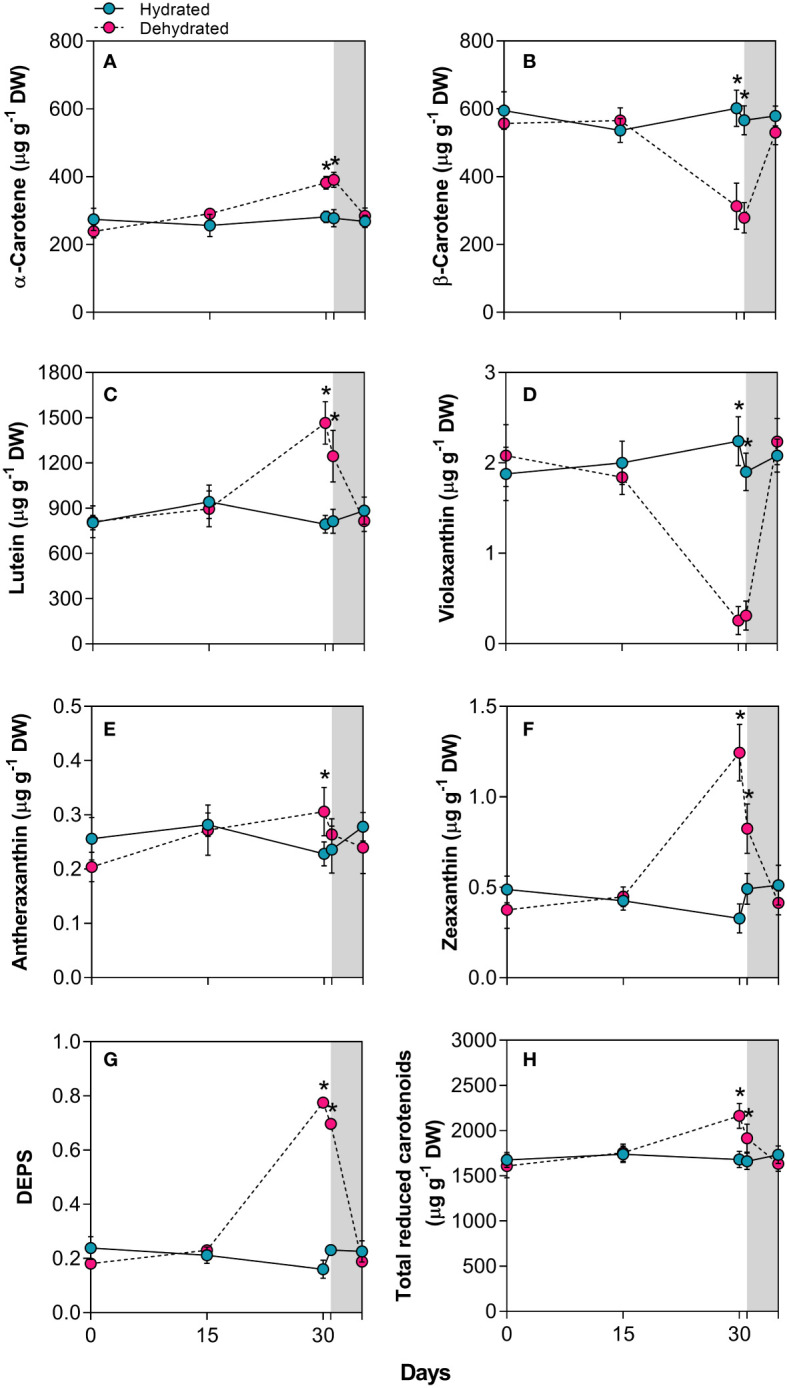
Carotenoids in leaves of *Barbacenia graminifolia* during the dehydration (0, 15 and 30 days) and after rehydration (24 and 120h). Leaf content of **(A)** α-carotene, **(B)** β-carotene, **(C)** lutein, **(D)** violaxanthin, **(E)** antheraxanthin, **(F)** zeaxanthin, **(G)** de-epoxidation state of the xanthophyll cycle – DEPS (*Z*+0.5 *A*/*V* + *Z* + *A*) and **(H)** total reduced carotenoids. The grey area in the figure indicates the rehydration period (between 30-35 days). *Significant difference between treatments at *P* < 0.05 (*n* = 5 ± *SD*).

The α-carotene concentration increased by 1.3 and 1.4 times in plants subjected to dehydration for 30 days and rehydration for 24 hours, respectively (**Figure 3A**). In contrast, β-carotene levels decreased significantly (p < 0.01) by approximately 1.9-fold and 2.0-fold in the same period (**Figure 3B**). As illustrated in **Figure 3C**, there were significant 1.8-fold and 1.5-fold increases in lutein concentration over the same period. The xanthophyll content also changed with stress imposition. While the violaxanthin concentration decreased drastically at 30 days, the antheraxanthin and zeaxanthin levels increased significantly, with a 3.7-fold increase for the latter (**Figures 3D–F**). The dynamics of xanthophyll adjustment led to an increase in the de-epoxidation state (DEPS) of the xanthophyll cycle (**Figure 3G**). This cycle involves the enzymatic de-epoxidation of violaxanthin (V) to zeaxanthin (Z) via antheraxanthin (A). The previously described increase in carotenoids led to a rise in total reduced carotenoids at 30 days (**Figure 3H**). After the resumption of water influx, both carotenoid and xanthophyll levels returned to values close to those of control plants 120h after rehydration.

### β-carotene cleavage and apocarotenoid increase during desiccation and recovery

**Figure 4:**
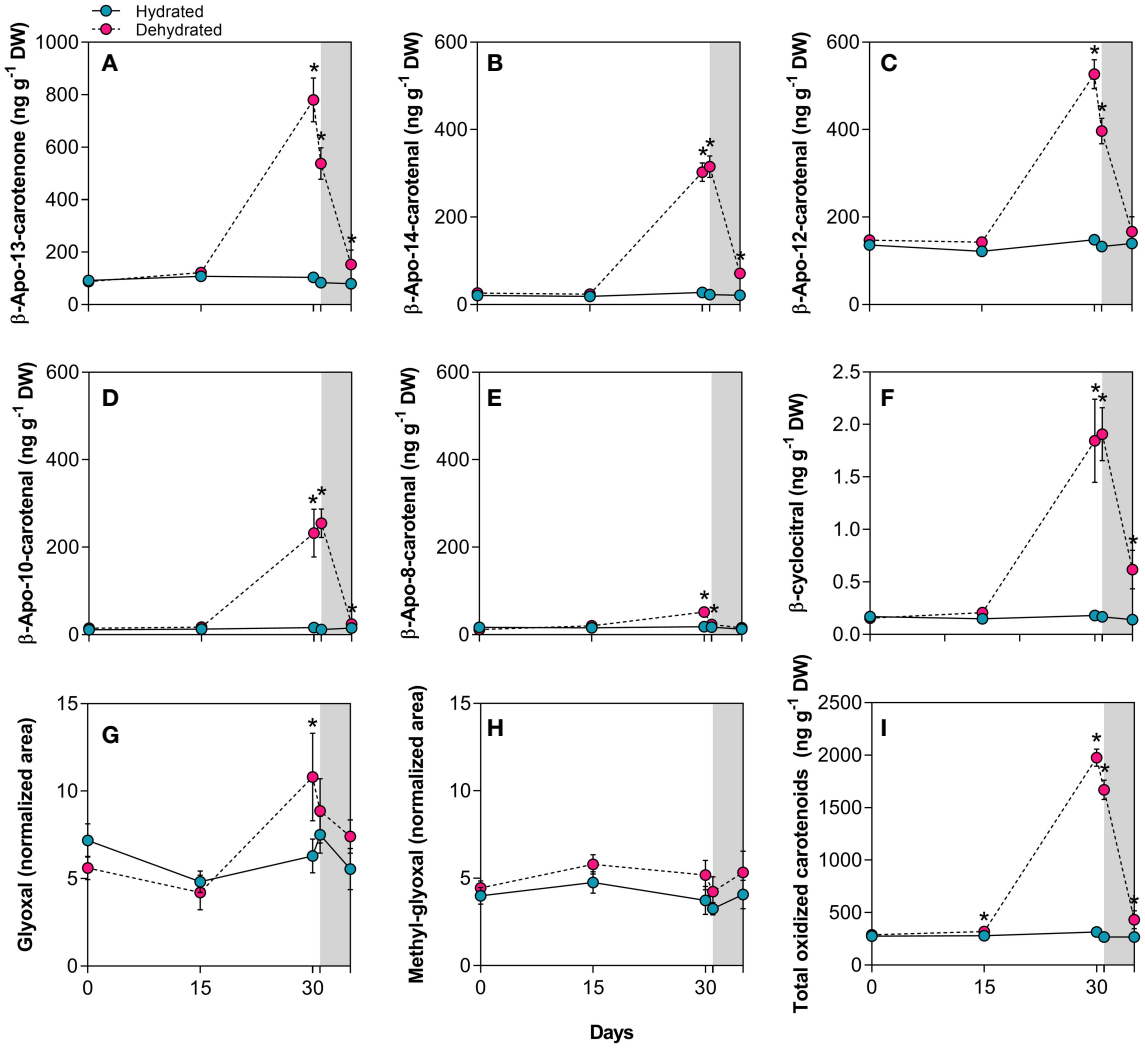
Apocarotenoids in leaves of *Barbacenia graminifolia* during the dehydration process (0, 15 and 30 days) and after rehydration (24 and 120h). Leaf content of β-apocarotenoids **(A–E)**, volatile cyclic β-cyclocitral **(F)**, glyoxal **(G)**, methyl-glyoxal **(H)** and total oxidized carotenoids **(I)**. β-apocarotenoids were determined by LC-MS analyses and expressed in ng g^-1^ DW, while glyoxal and methyl-glyoxal are expressed as peak areas normalized to internal standards and dry mass. The grey area in the figure indicates the rehydration (between 30-35 days). *Significant difference between treatments at *P*< 0.05 (*n* = 5 ± *SD*).

Apocarotenoid levels considerably increased during desiccation (**Figure 4**). The degradation of β-carotene in dehydrated *B. graminifolia* plants (**Figure 3B**) resulted in the production of several apocarotenoids with multiple functions. The most significant increases of β-apocarotenoids observed were: 14.1-fold for β-apo-10’-carotenal, followed by 10.9-fold for β-apo-14’-carotenal, 7.5-fold for β-apo-13-carotenone, 3.5-fold for β-apo-12’-carotenal and 2.8-fold for volatile β-apo-8’-carotenal at 30 days of stress imposition (**Figures 4A–E**). Another volatile β-apocarotenoid, the β-cyclocitral, showed a substantial increase in dried leaves, rising by 10.2-fold compared to hydrated plants at 30 days (**Figure 4F**). Similarly, apocarotene dialdehyde glyoxal increased 1.7 times during the same period, while methyl-glyoxal did not change significantly during monitoring (**Figures 4G, H**). These increases contributed to the overall elevation of total oxidized carotenoids (**Figure 4I**).

Interestingly, we detected increased concentrations of the apocarotenoids β-apo-13-carotenone, β-apo-14’-carotenal, β-apo-10’-carotenal and β-cyclocitral 24h after rehydration (**Figures 4A, B, D, F**). While most of the apocarotenoids reached levels close to the control after 120h, a maintenance of high levels was observed for β-cyclocitral (**Figure 4F**).

### Biosynthesis of tocopherols

**Figure 5:**
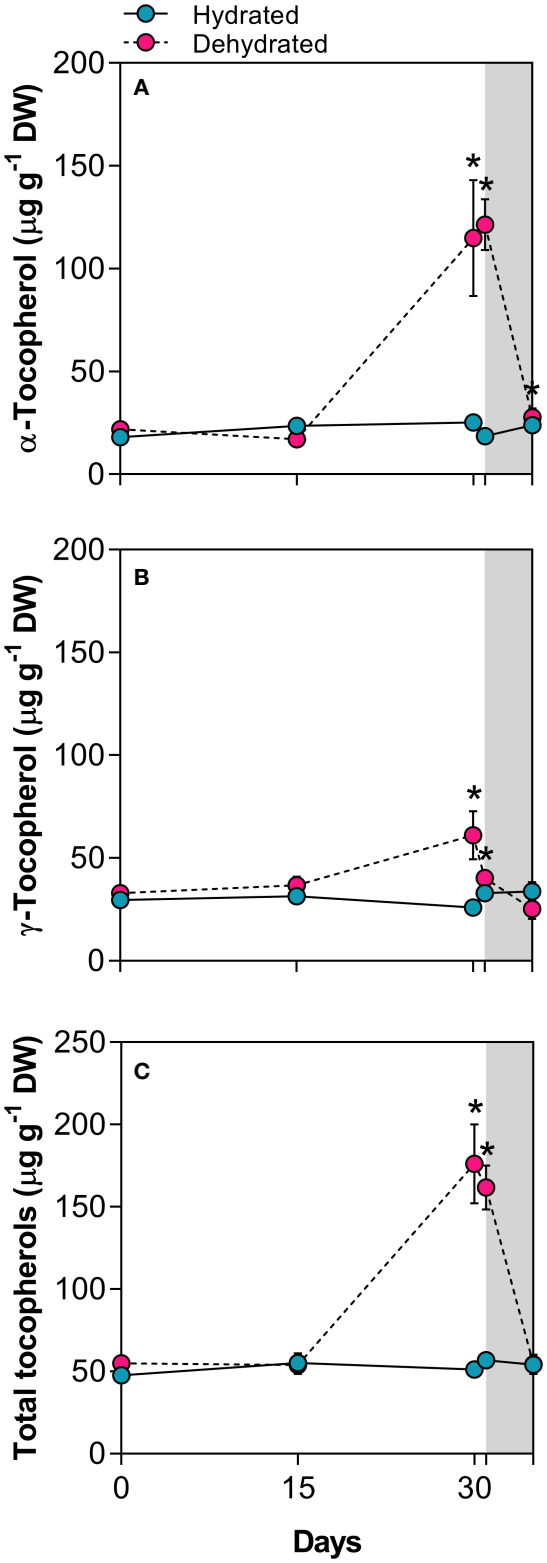
Tocopherols in leaves of *Barbacenia graminifolia* during the dehydration process (0, 15 and 30 days) and after rehydration (24 and 120h). Leaf content of **(A)** α-tocopherol, **(B)** γ-tocopherol, and **(C)** total tocopherols. The grey area in the figure indicates the rehydration period (between 30-35 days). *Significant difference between treatments at *P* < 0.05 (*n* = 5 ± *SD*).

The concentration of α-tocopherol significantly increased (4.6-fold) in dried leaves at 30 days and remained high (6.5-fold), even 24h after rehydration compared to control plants (**Figure 5A**). While levels of γ-tocopherol were lower than those of α-tocopherol, they also showed a significant increase, reaching 2.4-fold higher values in dried leaves (**Figure 5B**). As observed for other compounds, total tocopherol levels remained higher than those in control plants even 24h after rehydration (**Figure 5C**).

### Hormonal levels throughout dehydration-rehydration

**Figure 6:**
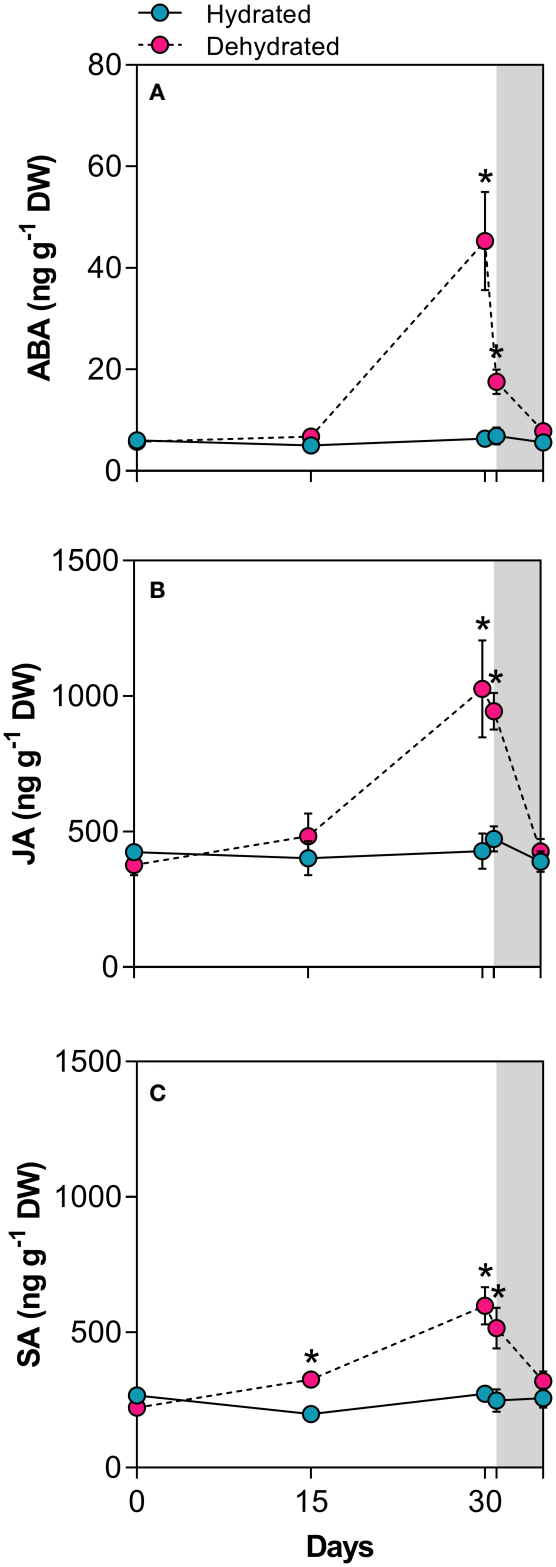
Levels of stress-related phytohormones in leaves of *Barbacenia graminifolia* during the dehydration (0, 15 and 30 days) and after rehydration (24 and 120h). Leaf content of **(A)** abscisic acid (ABA), **(B)** jasmonic acid (JA), and **(C)** salicylic acid (SA). The grey area in the figure indicates the rehydration period (between 30-35 days). *Significant difference between treatments at *P* < 0.05 (*n* = 5 ± *SD*).

Leaves of *B*. *graminifolia* exhibited basal hormonal levels under normal water supply. After 15 days of desiccation, ABA and JA concentrations remained close to the values of hydrated plants, while SA increased significantly during this period (**Figure 6**). At 30 days of dehydration, dried leaves showed a 7.1-fold increase in ABA levels and a 2.4-fold and 2.2-fold increases for JA and SA concentrations, respectively (**Figures 6A–C**). Although all hormone levels remained high at the beginning of rehydration (24h), a more pronounced reduction was observed in ABA levels (**Figure 6A**). After 120h of rehydration, all hormone levels returned to the basal levels observed in control plants (**Figure 6**).

### Principal component analysis

**Figure 7:**
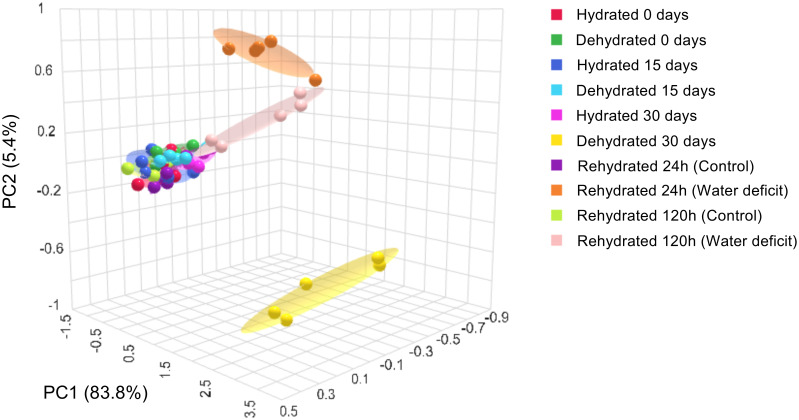
Principal components analysis (PCA) of the physiological and biochemical parameters in leaves of *Barbacenia graminifolia* during the dehydration (0, 15 and 30 days) and after rehydration (24 and 120h). See more in **Supplementary Table S1**.

Scores derived from principal component analysis were generated for both drought-stressed and non-stressed conditions (**Figure 7**). The score scatter plots of PCA indicated that the PC1 accounted for 83.8% of the total data variance, with PC2 contributing a modest 5.4%. The PCA revealed completely distinct groupings, where plants that were fully dehydrated at 30 days formed a separate cluster from both hydrated plants and those dehydrated for up to 15 days. It is noteworthy that at 24 and 120h post-rehydration, the grouping of the clusters approached the patterns observed for fully hydrated plants and those under stress for up to 15 days. This finding suggests an effective mechanism for the recovery from oxidative stress induced by severe dehydration. **Supplementary Figure S2** illustrates that the majority of antioxidant compounds, along with certain oxidation products of carotenoids, played a more significant role in clustering patterns for plants under dehydration.

**Figure 8:**
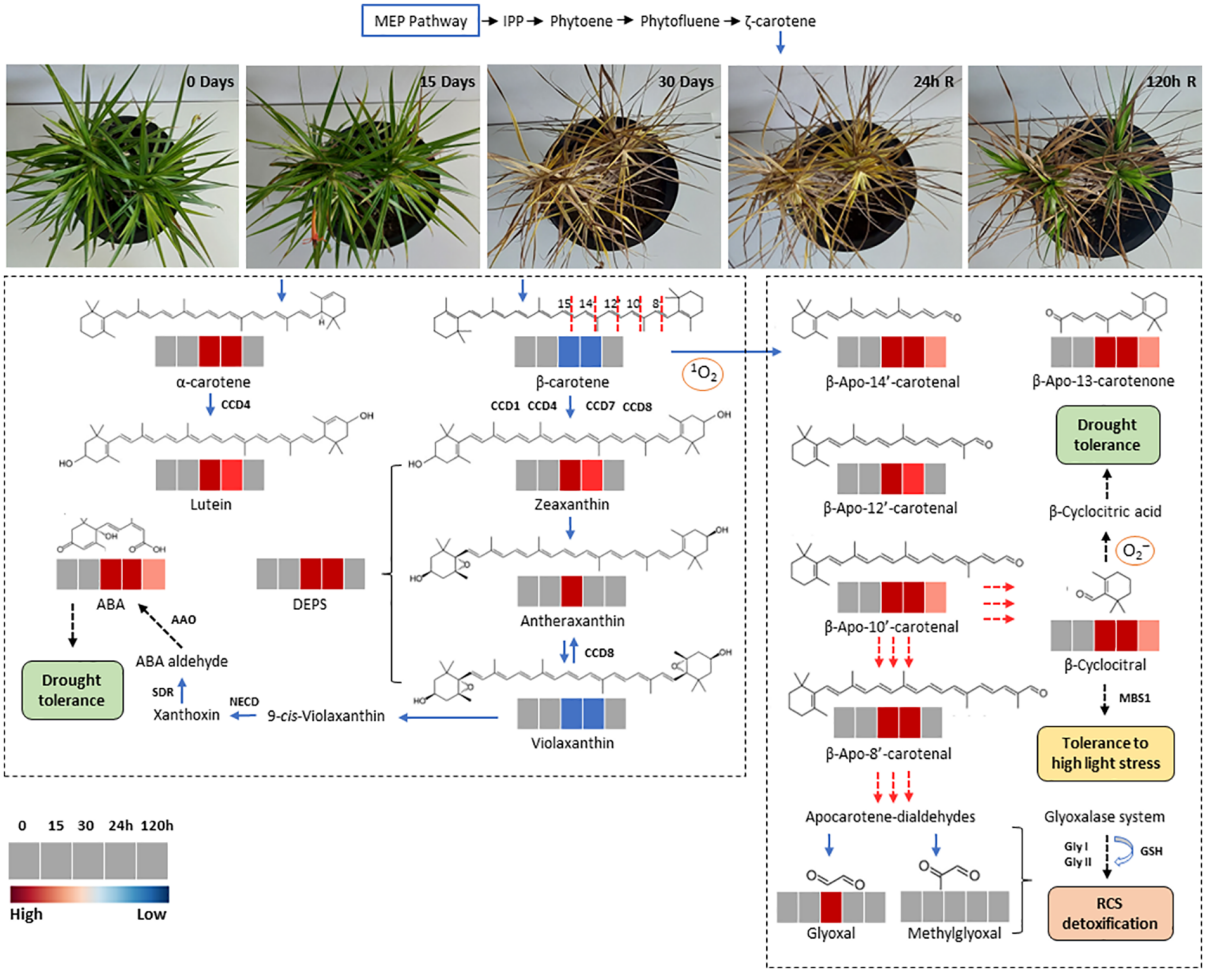
Schematic representation of carotenoid synthesis and β-carotene oxidation products (Apocarotenoids) in leaves of *Barbacenia graminifolia* during the dehydration (0, 15 and 30 days) and after rehydration (24 and 120h). The isopentenyl diphosphate (IPP) and other precursors derived from the methyl-d-erythritol 4-phosphate (MEP) pathway are indicated in the upper part of the figure. The red dashed lines indicate the reacting double bond position of β-carotene cleavage; the corresponding cleavage products are depicted in the right frame. Secondary oxidation results in the release of linear apocarotene-dialdehydes and β-cyclocitral. Methylglyoxal and glyoxal represent end products after continued oxidation of carotene dialdehydes. The dashed arrows indicate the formation of final compounds and their physiological effect. Enzymes are represented in bold. Colors represent the concentration of each compound (red, significantly higher than control; blue, significantly lower than control; grey, no significant changes at p < 0.05).

## Discussion

### ROS scavenging via ascorbate-glutathione cycle

As previously reported, *Barbacenia graminifolia* possesses photoprotective and antioxidative mechanisms that contribute to preserving desiccated tissues and maintaining their viability for recovery upon rehydration. This tolerance strategy involves the shutdown of photosynthetic metabolism and chlorophyll degradation, along with the production and degradation of antioxidant compounds (Vieira et al., 2022). The results presented here provide additional information supporting this presumed protective action through the increase of various antioxidant compounds. Despite the occurrence of ROS accumulation and membrane injury in *B*. *graminifolia* leaves, the antioxidant defense system appeared to be efficiently activated as indicated by the increased levels of Asc and GSH. The increase in these compounds, detected at 30 days of water suppression, persisted towards rehydration, suggesting that they may partially act to maintain ROS levels at tolerable or sublethal concentrations. In *Haberlea rodopensis*, Asc and GSH have been implicated in the prevention of photoxidation at very low RWC when photosynthetic activity is inhibited (Georgieva et al., 2017). Furthermore, the overexpression of several genes encoding ascorbate and glutathione peroxidases, as well as monodehydroascorbate and glutathione reductases, was reported in this species under dehydration (Gechev et al., 2013; Liu et al., 2018).

### Changes in carotenoid and xanthophyll levels improve photoprotection

Plant carotenoids play diverse roles, such as regulation of light harvesting and protection against photooxidative stress. Our study revealed a substantial accumulation of α-carotene after 30 days of dehydration, potentially linked to lutein biosynthesis, effectively contributing to the protection of the light-harvesting complexes (LHCs). Lutein derived from α-carotene constitutes approximately half of the total carotenoids in the chloroplasts. It accumulates in the thylakoids, participating in light harvesting and conferring photoprotection of the antenna complexes (Niu et al., 2020). Since *B. graminifolia* occurs in rocky environments exposed to high irradiance, the non-photochemical chlorophyll fluorescence quenching (NPQ) represents the most rapid and efficient mechanism to dissipate the excess photosystem II light energy into heat. According to Dall'Osto et al. (2006), triplet chlorophyll and singlet oxygen formation and accumulation are efficiently counteracted by increasing or converting α-carotene into lutein, facilitating the dissipation of excess energy from the xanthophyll cycle.

Conversely, β-carotene levels in *B*. *graminifolia* decreased markedly with increasing dehydration, consistent with previous findings in other resurrection plants (Farrant et al., 2003; Beckett et al., 2012; Rapparini et al., 2015; Georgieva et al., 2017). Our data does not allow us to infer whether the reduced levels of β-carotene reflect only a breakdown of PSII or if it could be an effective strategy against photoinhibition. Mor et al. (2014) report that carotenoid degradation occurs even at low chlorophyll levels, and singlet oxygen formation is not exclusive to photosynthesizing tissues but can also occur in chlorophyll-free tissues. Thus, continuous β-carotene degradation, even 24h after rehydration, may represent a strategy for *B*. *graminifolia* to minimize oxidative damage in the unrestored photosynthetic apparatus.

Concurrently with the maintenance of photosystem functionality, the decrease in violaxanthin and the increase in zeaxanthin levels via antheraxanthin interconversion appear to be important mechanisms for the dissipation of excess energy during *B. graminifolia* dehydration. Zeaxanthin plays a central role in NPQ since the quenching of excess absorbed light energy is transferred from chlorophylls to the chlorophyll-zeaxanthin heterodimer before being dissipated as heat (Sacharz et al., 2017; Sun et al., 2022). Thus, the observed zeaxanthin accumulation likely influenced the LHCII-PsbS protein interaction, which is essential in the NPQ photoprotection activity. According to reports by several authors (Munné-Bosch and Alegre 2000; Fernández-Marín et al., 2011; Verhoeven, 2014; Míguez et al., 2015; Verhoeven et al., 2018) in desiccation conditions, the high correlations between reduced Fv/Fm and elevated AZ/VAZ have led to hypotheses suggesting that sustained forms of xanthophyll cycle-associated thermal energy dissipation are engaged in such conditions, causing reductions in photochemical efficiency. Still, although photoinhibition occurs, *B. graminifolia* appears capable of safely regulating photosynthetic machinery activity during dehydration. Furthermore, the observed increase in the de-epoxidation state of the xanthophyll cycle (DEPS) in this study supports the notion that the xanthophyll cycle plays a more prominent role in physically quenching of excess energy through thermal dissipation. This mechanism could represent a protection for the photosynthetic apparatus against the risk of overexcitation throughout the dehydration-rehydration cycle.

### β-apocarotenoids roles and ROS detoxification during dehydration and carotenogenesis after rehydration

The dismantling of photosynthetic apparatus in the poikilochlorophyllous plant *B*. *graminifolia* likely results in the release carotenoids, increasing oxidative susceptibility and resembling senescence-mediated catabolism in the chloroplasts. In addition to β-carotene acting as an effective antioxidant, it is plausible that the products resulting from its cleavage may serve as precursors to other compounds that modulates antioxidant activity, thereby mitigating photooxidative stress (Zhu et al., 2023). Pear plants treated with β-CAA exhibited reduced ROS levels by increasing the activities of SOD and POD enzymes (Zhu et al., 2023). The identification of accumulated β-apocarotenals, including β-apo-13-carotenone, β-apo-14’-carotenal, β-apo-12’-carotenal and β-apo-10’-carotenal in *B. graminifolia* leaves coincides with β-carotene degradation after 15 days of dehydration. The sustained high levels of β-apo-13-carotenone, β-apo-14’-carotenal, β-apo-10’-carotenal and β-cyclocitral could represent an important protective mechanism against ROS excess 24h after water influx, and also for carotenoid resynthesis after the restoration of assimilatory metabolism. As previously suggested (D’Alessandro and Havaux, 2019; Deshpande et al., 2021b; Koschmieder et al., 2021), apocarotenoids play a role in regulating/modulating plant stress responses. Therefore, β-carotene cleavage in *B*. *graminifolia* leaves might be related to the direct defensive action of the byproducts formed against high light stress, as well as ROS scavenging. The CCD7 enzyme catalyzes the cleavage (9, 10; 9’,10’) of β-carotene into β-ionone and the C_27_ compound β-apo-10’-carotenal. CCD8 subsequently converts β-apo-10’-carotenal into C_18_ compound β-apo-13-carotenone (Dhar et al., 2020). These sequential cleavages by these plastid-localized enzymes generate the initial compound for strigolactone (SL) biosynthesis. Alvi et al. (2022) reported that SLs could modulate the ability of leaves to capture light energy by altering photosynthetic pigment components. Therefore, increases in β-apo-10’-carotenal and β-apo-13-carotenone in *B*. *graminifolia* leaves may indicate an effective control of excess light absorption and ROS generation during dehydration, possibly via the SL pathway.

Short-chain β-apocarotenoid like β-CC and the long-chain β-apo-8’-carotenal are generated by continued oxidative cleavage of β-apocarotenals, in line with the increased levels of these compounds observed in *B. graminifolia* during dehydration. The accumulation of β-CC and its conversion in β-CCA is activated under water deficit, osmotic stress and high irradiance, regulating the expression of singlet oxygen-responsive genes and eliciting gene expression patterns that enhance photooxidative stress tolerance (Deshpande et al., 2021a; Braat et al., 2023; Gómez-Sagasti et al., 2023). According to D'Alessandro et al. (2019), β-CC acts as a signaling molecule, inducing changes in the expression of a broad set of nuclear-encoded ^1^O_2_-responsive genes. Recent transcriptome analyses of β-CC-treated plants showed a possible interconnection between β-CC and 3-phosphoadenosine 5-phosphate (PAP) signaling pathway, where β-CC induces PAP accumulation in response to altered photosynthesis under drought and high light stress (Ramel et al., 2012). We hypothesize that during the dehydration of *B*. *graminifolia*, the increase in β-CC may lead to its conversion into β-CCA, resulting in a photooxidative defense. In peach plants, β-CCA positively modulated drought tolerance, which is mainly mediated by enhancing photosynthesis and reducing ROS (Zhu et al., 2023). The increase of β-apo-8’-carotenal observed in plants of *B. graminifolia* under dehydration, although significant compared to control, was much lower than observed for other β-apocarotenals suggesting its further metabolic conversion (Durojaye et al., 2019).

Secondary oxidation caused by dehydration in *B*. *graminifolia* generated apocarotene-dialdehydes and produced the potential cytotoxic end product glyoxal. The increase in glyoxal under dehydration may result from lipid peroxidation and β-carotene oxidation, as mentioned by Koschmieder et al. (2022). Indeed, even with the increase in glyoxal at 30 days, the effective control of methylglyoxal levels in the leaves suggests an efficient detoxification mechanism during the dehydration of *B*. *graminifolia*. Therefore, it is reasonable to suppose that the glyoxalase pathway might improve the detoxification of these compounds using GSH as a catalyst. The low level of violaxanthin detected at 30 days of dehydration coincided with the highest ABA level in *B. graminifolia*. These results suggest that carotenoid-derived phytohormone pathway might be an important mechanism for desiccation tolerance. As described by Zheng et al. (2021), violaxanthin and neoxanthin can be converted into 9-cis-violaxanthin and 9’-cis-neoxanthin by 9-cis-epoxycarotenoid dioxygenases (NCEDs) producing xanthoxin, which is then converted into ABA. The signaling-protection role of the ABA apocarotenoid has been well demonstrated in several resurrection plants (Rodriguez et al., 2010; Djilianov et al., 2013; Vieira et al., 2017a; Gu et al., 2019; John et al., 2023).

In *B*. *graminifolia* post-stress recovery (24-120h), the carotenogenesis process is corroborated by decreased apocarotenoid levels and β-carotene resynthesis. The great diversity of primary β-apocarotenoids derivatives, which are expected to be formed *in planta*, most likely represent intermediates that feed into subsequent metabolic routes, including glycosylations and esterifications (Koschmieder et al., 2021). Amongst these routes, the regulation of carotenogenesis by apocarotenoids in plant development and response to biotic and abiotic stress conditions has been proposed, irrespective of their metabolic origin (Pogson et al., 2008; Ohmiya, 2009; Hou et al., 2016; D'Ambrosio et al., 2023). However, the role of β-apocarotenoids in carotenogenesis in the post-stress recovery process in resurrection plants is still unclear.

Our findings indicate that the water influx after rehydration seems to be the starting point of the interconversion of apocarotenoids into carotenoids, especially β-carotene. Hou et al. (2016) emphasize that regardless of metabolic origin, apocarotenoids help finetune carotenogenesis for environmental responses and plant development. In leaves of monocotyledonous resurrection plants, the proplastid transformation into functional chloroplasts occurs during rehydration and the plastoglobuli, as lipid reservoirs, may assist in rapidly forming thylakoid membranes, supplying lipid building blocks for membrane restructuring (Ahrazem et al., 2019). As demonstrated by Vieira et al. (2022), the plastoglobules increase in the dehydration-rehydration cycle of *B*. *graminifolia.* Therefore, it is reasonable to suppose that chloroplast restructuring may be accompanied by β-carotene resynthesis, regulated by apocarotenoids during the post-stress recovery of *B*. *graminifolia*. This strategy may have a signaling role in the resynthesis of various compounds for metabolic recovery and a protective function against photooxidation. Thus, the regulation of carotenoid biosynthesis and its turnover to produce apocarotenoids appears to be tightly controlled to maintain stable levels during the dehydration-rehydration cycle (**Figure 8**).

### Hormonal interplay and cross-talk in the antioxidant regulation

Our results suggest an interconnection among ROS, antioxidants, and phytohormones in response to water deficit and high light stress. In *B*. *graminifolia*, the increased levels of ABA and JA after 30 days suggest that these stress hormones may act as master regulators of stomatal closure and protection of the photosynthetic apparatus. This likely involves the xanthophyll cycle-dependent excess energy dissipation, leading to increased desiccation tolerance. According to Müller and Munné-Bosch (2021), ABA plays a role in mediating the pigment pool of the xanthophyll cycle during water deficit, leading to increased NPQ. The authors emphasize that ABA signal detection and transduction are also closely linked to redox signaling, potentially disrupting the electron transport chain in chloroplasts. In *Myrothamnus flabellifolia*, components of the photosynthetic apparatus of ABA-induced genes, such as reaction center subunits, electron transport chain proteins, and ATP synthase, were repressed, likely to limit ROS generation and subsequent oxidative stress (Gechev et al., 2021).

Our findings provide correlative evidence that ABA and JA increase antioxidants associated to the ascorbate–glutathione cycle and tocopherols. As reported by Wu et al. (2018), ABA signaling can induce gene expression of the ascorbate peroxidase (APX2), a protein tightly distributed around chloroplasts, playing key roles in H_2_O_2_ homeostasis and chloroplast protection through H_2_O_2_ conversion into H_2_O in the ascorbate–glutathione cycle. Similarly, the specific regulation of vitamin E by ABA and JA during the dehydration of *B*. *graminifolia* enhances protection against photooxidation. Regulation of vitamin E biosynthesis by ABA and jasmonates has been proposed in several studies for other species (Sandorf and Holländer-Czytko, 2002; El Kayal et al., 2006; Siles et al., 2018; Villadangos and Munné-Bosch, 2023). The increase in α-tocopherol in thylakoids has been proposed to provide photoprotection by scavenging free radicals and ^1^O_2_, thereby exerting a synergic effect with other antioxidants. Additionally, photoprotection could be achieved by reducing thylakoid membrane permeability to protons, promoting the acidification of the thylakoid lumen under high irradiance and activating violaxanthin de-epoxidase (Munné-Bosch and Alegre, 2002). In support of this, Morales et al. (2014) demonstrated that the DT species *Vellozia gigantea* increased ABA and JA as part of a drought tolerance mechanism, with vitamin E levels serving as a crucial factor.

## Conclusions

Our results showed that *B*. *graminifolia* undergoes tight adjustments in its antioxidant metabolism in response to desiccation. These adjustments appear to be associated with processes directly or indirectly regulated by carotenoid degradation and biosynthesis, as well as the increase in α-tocopherol and compounds of ascorbate-glutathione cycle in the dehydration-rehydration cycle. Our data also suggest that apocarotenoids, carotenoid-cleavage products, may serve as regulators and precursors of protective compounds in leaves of *B*. *graminifolia* in response to variations of environmental water availability. This regulation, combined with other protective mechanisms previously described by our group, likely contributes to the success of this resurrection plant in its challenging habitat.

## Data availability statement

The raw data supporting the conclusions of this article will be made available by the authors, without undue reservation.

## Author contributions

EV: Conceptualization, Data curation, Formal analysis, Investigation, Methodology, Supervision, Validation, Writing – original draft, Writing – review & editing. MG: Data curation, Validation, Writing – review & editing, Investigation. CC: Data curation, Funding acquisition, Investigation, Validation, Writing – review & editing. SM: Conceptualization, Data curation, Investigation, Methodology, Supervision, Validation, Writing – review & editing. MB: Conceptualization, Data curation, Investigation, Validation, Writing – review & editing.

## Supplementary material

Supplementary Figure 1 Microclimate variables monitored daily throughout the experiment. **(A)** Bars indicate photosynthetic photon flux density (PPFD, μmol photons m^−2^ s^−1^) and continuous red lines correspond to average temperature (°C). In **(B)**, dark bars show the average values of relative humidity (RH%) and continuous blue line corresponds to the values of the vapor pressure deficit (VPD).

Supplementary Figure 2 Contribution of main parameters to cluster grouping from Principal Component Analysis (PCA) in leaves of *Barbacenia graminifolia* during dehydration (0, 15, and 30 days) and after rehydration (24 and 120h). The leaf content of antioxidant compounds (GSH, GSSG, α-tocopherol), ABA, most β-apocarotenoids, and volatile cyclic β-cyclocitral contributed to the grouping of the cluster in dehydrated plants at 30 days and 24 hours after rehydration. In hydrated plants, dehydrated for up to 15 days and rehydrated at 120h, RWC, β-carotene, and violaxanthin data were the most representative for cluster grouping. (See more in **Supplementary Table S1**).

Supplementary Table 1 The feature details of PCA analysis, showing the correspondence between data of the main evaluated parameters in leaves of *Barbacenia graminifolia* during dehydration (0, 15, and 30 days) and after rehydration (24 and 120h).
