# Supplementary figures and images for: Desiccation tolerance in the resurrection plant Barbacenia graminifolia involves changes in redox metabolism and carotenoid oxidation
Source: Front Plant Sci. 2024 Feb 15;15:1344820. doi: 10.3389/fpls.2024.1344820 (PMC10902171; doi:10.3389/fpls.2024.1344820)

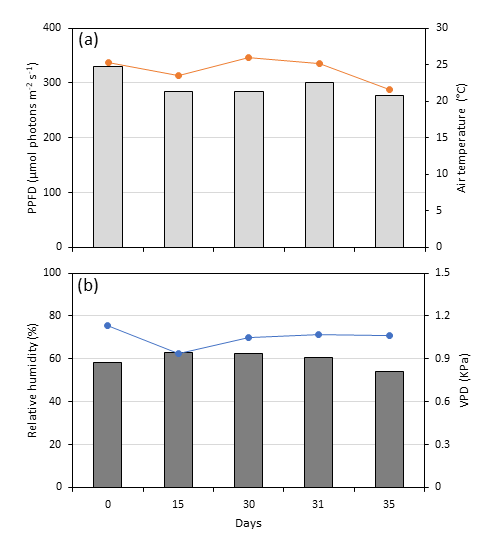

Supplement: Supplementary Figure 1 — Microclimate variables monitored daily throughout the experiment. (A) Bars indicate photosynthetic photon flux density (PPFD, μmol photons m−2 s−1) and continuous red lines correspond to average temperature (°C). In (B), dark bars show the average values of relative humidity (RH%) and continuous blue line corresponds to the values of the vapor pressure deficit (VPD). [file Image_1.tif]

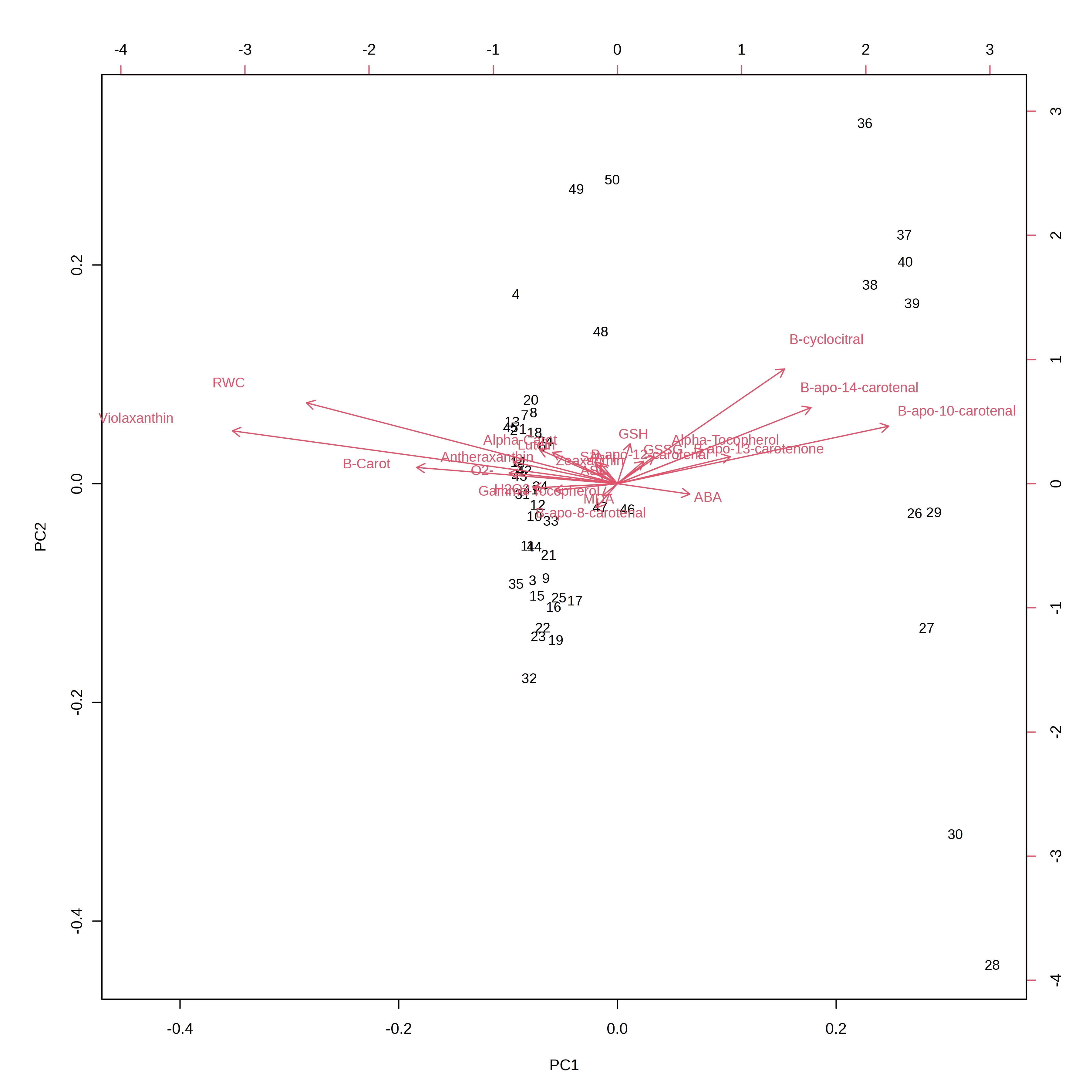

Supplement: Supplementary Figure 2 — Contribution of main parameters to cluster grouping from Principal Component Analysis (PCA) in leaves of Barbacenia graminifolia during dehydration (0, 15, and 30 days) and after rehydration (24 and 120h). The leaf content of antioxidant compounds (GSH, GSSG, α-tocopherol), ABA, most β-apocarotenoids, and volatile cyclic β-cyclocitral contributed to the grouping of the cluster in dehydrated plants at 30 days and 24 hours after rehydration. In hydrated plants, dehydrated for up to 15 days and rehydrated at 120h, RWC, β-carotene, and violaxanthin data were the most representative for cluster grouping. (See more in Supplementary Table S1 ). [file Image_2.tiff]
